# Understanding the Effects of Drishti Bheda, the Eye Movements in Indian Classical Dance, on Vision, Audition, and Cognition: A Cross-Sectional Study

**DOI:** 10.7759/cureus.117052

**Published:** 2026-09-28

**Authors:** Rakshitha Sundarrajan, Renu Meena M, Anu S, Rajasekran P, Ramji M

**Affiliations:** 1 Physiology, Velammal Medical College Hospital and Research Institute, Madurai, IND; 2 ENT, Velammal Medical College Hospital and Research Institute, Madurai, IND; 3 Community Medicine, Velammal Medical College Hospital and Research Institute, Madurai, IND

**Keywords:** bharatanatyam, cognition, drishti bheda, eye movements, neuroplasticity

## Abstract

Introduction: Bharatanatyam is one of India's oldest classical Indian art forms, which integrates the practice of Drishti Bheda, i.e., synchronized controlled scanning eye movements with head, neck, and body movements used to amplify visual articulation and communication. These ocular workouts repeated over time suggest that eye training might boost up information from sight and hearing to cognitive capacities, as well as control and ability to regulate motor imagery, which relates to one’s ability to see/feel movement without physical acting, to stimulate mental clarity, mindfulness, and boost emotions via plasticity of neural networks. Although Drishti Beda is artistically relevant to culture and the world of art, it lacks sufficient research to determine the impact on sensorial and cognitive skills. Knowledge of these effects can provide the scientific rationale to include traditional eye movement meditation exercises in preventive healthcare protocols for the brain and the senses and cognitive rehabilitation. This research assessed long-term Bharatanatyam training with Drishti Bheda on visual, auditory, and cognitive performance in healthy young individuals to understand if longer participation in the demanding nature of Bharatanatyam translates into improved sensory processing and overall cognition.

Methods: A cross-sectional comparative study was conducted in 60 healthy young females aged 18-22 years after obtaining Institutional Ethics Committee (IEC) clearance. Of the two groups involved, the 30 Bharatanatyam dancers had a formal training period of more than 10 years in dance, whereas their age-matched non-dancing counterparts formed the control group. Demographic and anthropometric assessments were performed on all subjects; the visual aspect of health was studied using critical flicker fusion frequency (CFFF-Maven Labs Pvt. Ltd., Bangalore, India), while the auditory aspect of health was determined using pure tone audiometry (PTA) (RS I digital portable diagnostic audiometer, Sounds Good Hearing Care Scientific Company, Madurai, India); the mental health aspects of health were assessed using the Mindful Attention Awareness Scale (MAAS), Movement Imagery Questionnaire-3 (MIQ-3), the Emotion Regulation Questionnaire (ERQ), and the Perceived Stress Scale (PSS-10). The groups were analyzed using independent-samples t tests.

Results: This study found that Bharatanatyam performers exhibited significantly higher CFFF values (visual temporal resolution; p < 0.0001) and PTA thresholds (auditory sensitivity) in the right ear (p = 0.0053) and left ear (p = 0.0006) compared with the control group. Similarly, statistically significant improvements in cognitive measures were observed in dancers in mindfulness (p≈0.0004), perceived stress (p < 0.0001), emotion regulation (p = 0.0013), and movement imagery (p < 0.0001).

Conclusion: Long-term Bharatanatyam training, along with Drishti Bheda, was associated with improved visual, auditory, and cognitive abilities in young adult brains. It is likely that the multisensory-training aspect of the performance might involve a variety of neural modifications or plasticity across and within different sensory modalities, and different functional brain areas or systems, involved with attentional control, motor imagination, and emotion-processing abilities.

## Introduction

Bharatanatyam is a traditional form of Indian dance that portrays complex facial expressions, hand mudras, and physical gestures [1]. In Bharatanatyam, eyes play a crucial role in conveying emotions and narratives, and this aesthetic appeal is known as "Drishti”. The focus and expression of the eyes are trained and choreographed to enhance the storytelling and emotional depth of the dance [2].

Derived from the Natyashastra, Drishti Bheda refers to the conscious usage of eye movements ranging from Sama, which is steady fixation, to elaborate searches in various directions, forming an inseparable part of the Bharatanatyam tradition. The eyes in dance have the crucial function of narrating, linking, and guiding the body towards the flow of expressions. Drishti Bheda is a sustained regimen of ocular motor training that repeatedly engages visual attention, temporal sequencing, and sensorimotor coordination.

Contemporary research places dance as a powerful multisensory intervention capable of driving structural and functional neuroplasticity, improving perceptual and executive processes across the lifespan [3]. Systematic reviews of dance-based training report changes in brain connectivity, motor planning, and cognitive performance, positioning dance as a model for integrative sensorimotor learning. Within the specific realm of Indian classical dance, eye-tracking studies have shown that expert Bharatanatyam observers and practitioners demonstrate [4] characteristic eye movement patterns and refined attentional allocation compared with non-dancers, implying that genre-specific visual training reshapes gaze behavior. Likewise, auditory studies in trained Bharatanatyam dancers report enhancements in auditory working memory and temporal processing, suggesting that prolonged musical motor engagement refines auditory pathways [5].

Despite these advances, a focused investigation that simultaneously evaluates visual temporal resolution, auditory sensitivity, and complementary cognitive domains within the same cohort of long-term classical dancers has not been published. Prior work tends to examine either passive eye-movement patterns during observation or isolated auditory/cognitive endpoints; comprehensive, multimodal assessments that link a culturally defined visual training practice (Drishti Bheda) with objective sensory thresholds and cognitive function remain lacking. Our study fills this gap by assessing critical flicker fusion frequency (CFFF) as a marker of temporal visual processing, pure tone audiometry (PTA) to quantify auditory thresholds, and validated cognitive instruments (attention, stress, emotion regulation, and motor imagery) in trained Bharatanatyam dancers versus matched controls--all within a single cross-sectional framework.

There are two rationales behind choosing CFFF: First, CFFF provides a sensitive, well-characterized index of visual temporal resolution, cortical arousal, and perceptual processing speed, with an established literature documenting its utility and methodological considerations [6]. Measuring CFFF in dancers allows direct probing of whether habitual, rhythmically paced visual training translates to measurable enhancements in fundamental visual temporal thresholds. Second, PTA was selected not merely as a clinical screen but as an objective window into auditory discrimination and temporal acuity, which are continuously engaged during dance through complex rhythmic entrainment and synchronized movement. Previous reports linking dance training to improved auditory temporal processing support this choice [7].

Cognition is an essential third pillar in our design. Dance training requires sustained attention, efficient working memory, motor imagery, and adaptive emotion regulation. These higher-order functions are supported by distributed neural networks, including the action observation network and frontoparietal attentional systems [8]. Integrating cognitive questionnaires (mindful attention, perceived stress, emotion regulation, and movement imagery) with sensory thresholds enables us to examine whether sensory enhancements co-occur with measurable cognitive advantages in attention, emotional control, and imagery, a conjunction that few studies have tested within the same participants. Broader reviews of dance and cognition indicate promising effects on executive function and attentional control, reinforcing the importance of measuring cognition alongside sensory metrics [9].

In summary, this research uses a well-rounded, culturally relevant approach to explore whether the focused eye practice of Drishti Bheda, within long-term Bharatanatyam training, is linked to improvement in visual resolution, auditory sensitivity, and cognitive function. By aggregating CFFF, PTA, and validated cognitive scales in a carefully chosen group of experienced dancers and controls, the study provides a new look at how a traditional visual practice might influence sensory processing and cognitive structure. This has implications for performance science and preventive wellness, and there are possible uses in sensory and motor rehabilitation.

## Materials and methods

This is an observational cross-sectional study that was conducted in the Departments of Physiology and Otorhinolaryngology at Velammal Medical College Hospital in Madurai, over a period of two months (July-August 2024), after getting approval from the Institutional Ethics Committee. The study followed the ethical standards cited in the Declaration of Helsinki, and informed consent was obtained from every participant before inclusion.

Participants were recruited using a screening questionnaire from students at the dance school, Shri Kalakshethram-Alayam of Music, and non-dancers from the same school who came to learn vocals. Those who met the eligibility criteria and provided written informed consent were included in the study. A total of 60 female participants were enlisted through convenience sampling based on a priori power analysis using G*Power software (Cohen's d = 0.8, α = 0.05, power = 80%), which showed a minimum requirement of 25 participants per group. Participants were divided into two groups: Group A comprised 30 trained Bharatanatyam dancers with a minimum of 10 years of formal training, while Group B included 30 age-matched healthy non-dancers with no history of dance or eye-movement training.

The inclusion criteria for the dancer group were females aged 18-22 years with a normal body mass index (BMI) who were actively practicing Bharatanatyam for a minimum of three to five hours a week, with Drishti Bheda as part of their training. The control group included healthy non-dancer females with the same age group and body mass index (BMI). The mean age of dancers was 18.93, and non-dancers was 19.00. The participants with refractive errors, eye and ear pathologies, hearing issues, neuromuscular issues, psychiatric illnesses, thyroid issues, or taking medications that interfere with the eyes, ears, or brain were not included in the trial. The visual, auditory, and cognitive functions were assessed using standardized, non-invasive tools.

Measurement of critical flicker fusion frequency (CFFF)

The critical flicker fusion frequency (CFFF), an light-emitting diode (LED) flicker fusion meter, namely the CFF M1 Model, manufactured by Maven Labs Pvt. Ltd., Bangalore, India, was used. A monochromatic light, especially red light (light-emitting diode) with a wavelength of 630 nm, was used, as it is perceived for a longer time in the retina [6]. The examination room was kept partially illuminated, the subject was made to sit comfortably, and a source of red light was presented at 25-30 cm. Gradually increase the frequency of oscillations. The subject was instructed to respond when the flickering light source appeared as a single fused light, and that frequency is the critical flicker fusion frequency. Netra software (Mavom Labs Pvt. Ltd., Bangalore, India) was used to record the frequency data. CFF was measured in both eyes separately, and the average of the two frequencies was considered the final CFF value for that subject [10].

Measurement of field of vision

Visual field measurement was done using the Priestley Smith's Perimeter (Rolex Scientific Engineers, Ambala, India) [11]. To record the visual fields of individual eyes using the perimeter, the subject was asked to place her chin on the perimeter stand and focus on the fixed point. The calibrated metal arc with a colored objective is moved till the objective is in view while focusing on the fixed point. The degree at which the objective is visualized is plotted on the revolving chart. The normal field of vision with a white object of 5 mm size is temporal 100 degree, since there is no anatomical obstruction on the temporal side of the eyes, the extent of the field of vision in this quadrant is more, inferior is 75 degree (maxilla of the cheek provides anatomical obstruction and reduces the extent of the field in this quadrant), superior 60 degree (supraorbital margin provides anatomical obstruction and reduces the extent of the field of vision in this quadrant), nasal 60 degree (nasal bridge provides anatomical obstruction and reduces the extent of the field of vision in this quadrant). The maximum degree obtained in the quadrant is used for further evaluation.

Measurement of pure tone audiometry

Auditory sensitivity was assessed using pure tone audiometry with a Graphic RS I digital portable diagnostic audiometer (Sounds Good Hearing Care Scientific Company, Madurai, India). Testing was performed in a sound-treated room using air conduction, with pure tones delivered separately to each ear through headphones at 250, 500, 1000, 2000, and 4000 Hz [12]. The audiometer was calibrated as per standard protocol prior to testing. Thresholds were obtained using the ascending method, in which tone intensity was increased in 5 dB steps until a consistent response was obtained; the subject was instructed to raise a hand when the tone was heard. Thresholds are expressed in decibels hearing level (dB HL) at each frequency.

Measurements of cognitive functions

Validated questionnaires were used to test cognitive function. These included the Mindful Attention Awareness Scale (MAAS) for cognitive attention, the Perceived Stress Scale (PSS) for stress levels, the Emotion Regulation Questionnaire (ERQ) for cognitive reappraisal and expressive suppression techniques, and the Movement Imagery Questionnaire (MIQ) for motor imagery. We have obtained formal permission to use the MAAS, ERQ, and MIQ-3 scales. The administration of the surveys took place in a calm setting, and scoring was done in accordance with recognized protocols.

Mindful Attention and Awareness Scale (MAAS) was developed by Brown and Ryan and is considered one of the most empirically supported measures of trait mindfulness--or one's general predisposition to pay attention and to be aware of the present experience during day-to-day life. The MAAS is a 15-item scale that is treated as a unidimensional scale, with higher scores representing increased mindful attention and awareness [13]. For each item of the questionnaire, answer options from 1 (almost always) to 6 (almost never) on a 6-point Likert scale are available. MAAS scale sum is the mean of the fifteen questionnaire items, a value that ranges between 1 and 6. A value of 1 means maximum mindful attention and awareness; a value of 6 refers to a minimal one. Thus, higher values (4.7 to 6.0) represent high levels of dispositional (trait) mindfulness. Lower values (1.0 to 3.3) denote low levels of mindful attention, or higher tendencies toward mindlessness. The total raw score ranges from 15 to 90.

While the Perceived Stress Scale (PSS-10) is an extensively used construct that evaluates perceptions of acute stressors, its properties in relation to individual stable (between-person) as versus transient (within-person) states are not well understood. It estimates how much variability is present over occasion on two repeated measures on the PSS and can be accounted for as between-person variance vs. within-person variance, respectively, and among various cohorts/population types. It is a 10-item version, the most widely used in research due to its good reliability and validity [14]. The PSS-10 is a 10-item self-report measure of the extent to which the situations in one's life are appraised as stressful. Individuals responding to the PSS-10 evaluate each item using a 5-point Likert scale ranging from 0 (never) to 4 (very often). Items 4, 5, 7, and 8 are reverse-scored and then summed together for a total score ranging from 0 to 40, which reflects the degree of perceived stress. Low stress (0 to 13) indicates low perceived stress, which means that you feel that you have good control over how you handle life's challenges. Moderate perceived stress (14-26) means that you feel you have some control over your ability to cope with life's challenges, but you need to develop better coping strategies. High stress (27 to 40) indicates high perceived stress, which means that life's demands may appear to be too great and overwhelming for you to cope with, which may have a detrimental impact on your day-to-day life.

The Emotion Regulation Questionnaire (ERQ) is a self-report tool that is used a lot to look at how people manage their emotions. Created by Gross and John, the ERQ has 10 questions that assess two ways of dealing with emotions. One way is called reappraisal, which is a way to change how someone sees a situation to make the feelings less strong. The other way is called suppression, which is about stopping emotions from showing on the outside [15]. The ERQ includes 10 items assessed on a 7-point Likert scale ranging from 1 (strongly disagree) to 7 (strongly agree). Two subscales assess emotion-regulation strategies: cognitive reappraisal (6 items; 1, 3, 5, 7, 8, 10) and expressive suppression (4 items; 2, 4, 6, 9). Scores can be calculated separately for the two subscales. Scores on cognitive reappraisal range from 6 to 42, and scores on expressive suppression range from 4 to 28. The higher the score, the greater the degree to which the individual reports using that particular emotion-regulation strategy. There are no reverse-keyed items in the original ERQ.

The Movement Imagery Questionnaire-3 (MIQ-3) is a widely used self-report measure for evaluating an individual's ability to produce mental images of movement. It studies three kinds of imagery: internal visual imagery, external visual imagery, and kinesthetic imagery. MIQ-3 has a total of 12 items relating to movement imagery in three dimensions, each with 4 items for that subscale. A 7-point scale from 1 (very difficult to see/feel) to 7 (very easy to see/feel). The total score range is from 12 to 84. The easier you can imagine/feel the movement imagery, the higher you would score in relation to that item. This makes the MIQ-3 a reliable and valid tool to evaluate imagery ability in sports, rehabilitation, and cognitive research [16].

Before the test was conducted, data regarding the individual's anthropometrics and demographics, for example, age, height, weight, and BMI, were taken. In a simultaneous activity that lasted around 30 minutes, all the tests were accomplished by the participant. To ensure the confidentiality of the participants, access to the records of the study was restricted, and data were anonymous throughout the investigation.

The Statistical Package for the Social Sciences (SPSS) (IBM Corp., Armonk, New York, USA) was used to analyze the data. The Shapiro-Wilk test was used to determine whether the data distribution was normal. The independent-samples t-test for normally distributed variables and the Mann-Whitney U test for non-normally distributed variables were used for intergroup comparisons. Statistical significance was defined as a p-value of less than 0.05.

## Results

The mean age of dancers was 18.93, and non-dancers was 19 (Table 1). Mean CFFF scores were observed to be higher among Bharatanatyam dancers when compared with the non-dancers. Statistically, mean CFFF values showed a significant difference in the right eye as well as in the left eye between dancers and non-dancers (p < 0.0001) (Table 2).

**Table 1:** Sociodemographic characteristics of dancers and non-dancers.

Sociodemographic variable	Dancers (n = 30)	Non-dancers (n = 30)	Total (N = 60)
Age (years), Mean ± SD	18.93 ± 0.52	19.00 ± 0.64	18.97 ± 0.58

**Table 2:** Comparison of CFFF values between dancers and non-dancers (visual domain). Independent (unpaired) t-test. CFFF: critical flicker fusion frequency. ***p < 0.001 = significant after Bonferroni correction (adjusted threshold p < 0.0036).

CFFF	Dancers (n = 30)	Non-dancers (n = 30)	t-value	p-value
Right eye	32.70 ± 3.22	26.33 ± 2.00	9.204	<0.0001***
Left eye	33.23 ± 3.48	24.50 ± 1.73	12.304	<0.0001***

The difference between groups was statistically significant in the right ear (p = 0.0053) but not after Bonferroni correction, and in the left ear (p = 0.0006), with significant variation in PTA threshold (dB HL) (Table 3).

**Table 3:** Comparison of PTA threshold values (dB HL) between dancers and non-dancers (auditory domain). Independent (unpaired) t-test. PTA: pure tone audiometry; dB HL: decibels hearing level. **p < 0.01 = highly statistically significant; ***p < 0.001 = very highly statistically significant. PTA in the right ear is not significant after Bonferroni correction (adjusted threshold p < 0.0036); ***p < 0.001 for the left ear remained significant.

PTA threshold (dB HL)	Dancers (n = 30)	Non-dancers (n = 30)	t-value	p-value
Right ear	16.14 ± 2.40	19.66 ± 6.19	−2.904	0.0053**
Left ear	15.32 ± 0.65	17.66 ± 3.47	−3.630	0.0006***

Comparison of visual field between dancers and non-dancers showed Bharatanatyam dancers having significantly greater field of vision in the inferior and temporal meridians of both eyes. For the left eye, the difference was statistically significant for the inferior (p < 0.001) and temporal (p < 0.001) meridians, and there was no significant difference between groups for the superior (p = 0.078) and nasal (p = 0.684) meridians (Table 4).

**Table 4:** Comparison of the field of vision of the left eye between dancers and non-dancers. Field of vision is expressed in degrees (°) as Mean ± SD. Independent (unpaired) t-test was used. Meridians (superior, inferior, nasal, and temporal) were prespecified secondary outcomes. ***p < 0.001 = significant after Bonferroni correction (adjusted threshold p < 0.0036).

Meridian	Dancers (n = 30), Mean ± SD (°)	Non-dancers (n = 30), Mean ± SD (°)	t-value	p-value
Superior	69.18 ± 2.02	70.12 ± 2.01	-1.807	0.078
Inferior	79.86 ± 2.84	71.18 ± 2.56	12.434	<0.001***
Nasal	60.47 ± 2.08	60.71 ± 2.42	-0.412	0.684
Temporal	99.54 ± 2.81	89.68 ± 3.18	12.726	<0.001***

In the right eye, results showed that the inferior (p < 0.001) and temporal (p < 0.001) meridians had significantly higher field of vision values in dancers as compared to non-dancers. No difference between the two groups was found for the superior (p = 0.071) and nasal (p = 0.850) meridians (Table 5).

**Table 5:** Comparison of the field of vision of the right eye between dancers and non-dancers. Field of vision is expressed in degrees (°) as Mean ± SD. Independent (unpaired) t-test was used. Meridians (superior, inferior, nasal, and temporal) were prespecified secondary outcomes. ***p < 0.001 = not significant after Bonferroni correction (adjusted threshold p < 0.0036).

Meridian	Dancers (n = 30), Mean ± SD (°)	Non-dancers (n = 30), Mean ± SD (°)	t-value	p-value
Superior	69.41 ± 1.96	70.34 ± 1.96	-1.838	0.071
Inferior	80.09 ± 2.90	70.97 ± 2.42	13.225	<0.001***
Nasal	60.39 ± 1.94	60.50 ± 2.48	-0.191	0.850
Temporal	99.79 ± 2.75	89.32 ± 3.24	13.494	<0.001***

Differences were observed across all questionnaires, with the mean MAAS score it was significantly higher among dancers when compared to non-dancers (P ≈ 0.0004). The mean PSS score showed a significantly lower value among dancers over non-dancers (P < 0.0001) and significantly higher values of ERQ scores among dancers (P = 0.0013), but not after Bonferroni correction, while a significantly higher MIQ score among dancers when compared to non-dancers (P < 0.0001). All these findings clearly indicated the presence of a significant difference in terms of different questionnaire-based cognitive measures between both groups (Table 6).

**Table 6:** Comparison of cognitive function between dancers and non-dancers using standardized questionnaires. Scores are expressed as Mean ± SD. Independent (unpaired) t-test was used. **p = 0.0013 for ERQ did not remain significant after Bonferroni correction (adjusted threshold p < 0.0036); ***p < 0.001 remained significant after correction. MAAS: Mindful Attention and Awareness Scale; PSS: Perceived Stress Scale; ERQ: Emotion Regulation Questionnaire; MIQ: Movement Imagery Questionnaire.

Questionnaire	Dancers (n = 30), Mean ± SD	Non-dancers (n = 30), Mean ± SD	t-value	p-value
MAAS	4.53 ± 0.59	3.88 ± 0.72	3.825	0.0004***
PSS	19.83 ± 3.05	25.66 ± 5.20	-5.297	<0.0001***
ERQ	4.55 ± 0.89	3.83 ± 0.73	3.426	0.0013**
MIQ	5.20 ± 0.34	4.54 ± 0.11	10.116	<0.0001***

## Discussion

In this study, trained Bharatanatyam dancers demonstrated significantly higher critical flicker fusion frequency (CFFF) values compared to non-dancers, reflecting improved visual temporal resolution and quicker visual information processing in the trained dancers (Table 2). The result indicates that the practice of rhythmic and gaze-targeted activities may be associated with greater visual temporal resolution of the visual system, which may index the underlying neural mechanisms facilitating efficient visual attention and rapid visual perceptual discriminations. The CFFF scores are well-known to be a sensitive index reflecting visual temporal processing and cortical arousal, which represent the rate at which the human visual system integrates discrete visual signals [6].

Numerous studies on dance expertise indicate that compared to novices, experts experience shorter fixation durations and tighter clustering of fixations while viewing complex movement sequences, which reflects greater control of attention and ability to identify relevant visual content [4,16]. Many of these previous studies focused on measuring eye movements using eye tracking while observers watched video recordings of dancer's movements, rather than directly assessing the observer's current level of visual temporal resolution, for example, via a contrast sensitivity function as measured by CFFF. In addition, studies of sensorimotor expertise support the idea that, through extensive training, an athlete's or dancer's sensitivity to biologically based motion increases, and that all individuals' sensitivity to biological motion is also enhanced through neurophysiological responses to actions reflected in visual processing [17,18]. This indicates that experience can change visual pathways, affecting how we process time and detect flicker. Unlike earlier studies that looked at general dance skills or eye movement patterns, this study specifically focuses on the importance of regular eye movement practice in enhancing visual processing. This finding could be significant for sensory training and neurorehabilitation.

The study also shows that trained Bharatanatyam dancers had significantly lower pure tone audiometric thresholds than non-dancers (Table 3). This indicates that dancers showed lower auditory thresholds, indicating greater auditory sensitivity. This difference may be related to prolonged exposure to complex rhythms, different tones, and regular practice of auditory motor synchronization as a part of classical dance training, though this cannot be confirmed by the present design [7].

Previous studies have shown that Bharatanatyam dancers have better auditory working memory. This suggests that their experience can change the auditory system, may be due to improved attention control and contralateral inhibition in the auditory pathway. However, these studies mainly looked at higher-level auditory thinking instead of measuring objective auditory sensitivity [5]. This study adds to other existing research by showing that training-related changes in hearing are seen not just in cognitive auditory tasks but also in basic auditory threshold measures from pure tone audiometry. This finding indicates that spending time with rhythm-based music and movement can improve both peripheral hearing sensitivity. This happens when we repeatedly activate the brain networks for hearing. It also leads to stronger connections between hearing and movement [19]. Unlike previous studies that looked at auditory memory or timing alone, this study combines auditory threshold assessment with visual and cognitive measures. It highlights the training in Bharatanatyam, mainly in the exercise of Drishti Bheda, as an important multisensory knowledge that can influence our auditory processing on both sensory and cognitive dimensions.

The present study shows significantly higher self-reported levels of mindful attention, adaptive emotion regulation, and motor imagery ability, along with lower perceived stress, among trained Bharatanatyam dancers when compared with non-dancers (Table 6). The higher scores observed may be related to sustained attentional and imagery demands of dance training [1]. Repeated practice in these processes promotes experience-dependent neuroplasticity in frontoparietal attentional networks, limbic regulatory circuits, and the action observation network. This network integrates perception, action planning, and emotional processing [8]. Studies have shown that dance training is linked to better executive function, attention, emotional control, and cognitive flexibility. This supports the idea that dance is a mentally enhancing activity [9,20]. Research specific to Bharatanatyam has also found that dancers have improved auditory working memory and attention control [5]. This suggests that training in classical dance boosts complex cognitive processing through rhythmic and sensorimotor involvement. However, most studies have looked at cognitive outcomes either separately or in the context of therapeutic dance programs, without evaluating sensory processing at the same time.

What is special about this study is that not only did we look at cognition but also included visual and auditory aspects of the human senses. What our study shows is that beyond cognitive skills, trained dancers also possess a greater capacity for sensory processing. We used well-established, tried-and-tested measuring tools, building on previous research, and found a direct link between the intensive visual engagement demanded by Drishti Bheda in Bharatanatyam training and enhanced attention, emotional self-control, and motor imagination in the participants.

In contrast to earlier studies that look at general exposure to dance, these findings show that Indian classical dance is a complete training program. It involves multiple senses and thinking processes that can possibly help with cognitive health and neurorehabilitation [21]. Since this is a cross-sectional study, self-selection cannot be ruled out, as dancers may have continued training partly due to pre-existing visual-motor or auditory ability. Other factors, such as physical activity, musical background, lifestyle, and socioeconomic status, could also have contributed to the differences observed.

The study had several limitations. It included only young female Bharatanatyam dancers aged 18 to 22. Therefore, the findings may differ for older or male Bharatanatyam dancers, or for those younger than the study participants. The study is limited in that it examined only one form of Indian classical dance--Bharatanatyam. Hence, the findings may not apply to other forms of dance. The current study is an example of a correlational study and does not provide evidence of causality. Another limitation is that the number of participants in the study is small, and participants came from the same background, which diminishes the relevance of the findings. Additionally, the ERQ was analyzed using a combined score rather than its two subscales (cognitive reappraisal and expressive suppression), as item-level data required for subscale calculation were not retained. This limits interpretation of the specific emotion-regulation strategy reflected in this finding.

## Conclusions

Long-term Bharatanatyam practice, including Drishti Bheda training, was associated with more favorable visual, auditory, and cognitive outcomes in healthy young individuals. Bharatanatyam dancers present with much greater CFFF values, lower PTA thresholds, increased mindfulness, enhanced motor imagery, better emotion regulation, and reduced stress levels. All of the above-mentioned facts indicate that these differences may reflect neuroplastic adaptation, though this requires confirmation through a longitudinal study.

Therefore, Bharatanatyam can be regarded as a non-pharmacological technique for the enhancement of sensory and cognitive functions. However, longer-term and multidisciplinary studies using brain-imaging techniques with a larger group are needed for more logical reasoning and to understand the neurobiological mechanisms underlying this phenomenon.
